# The mediating effect of psychological resilience between social support and anxiety/depression in people living with HIV/AIDS–a study from China

**DOI:** 10.1186/s12889-023-17403-y

**Published:** 2023-12-08

**Authors:** Yongbing Sun, Bing Song, Cheng Zhen, Chao Zhang, Juan Cheng, Tianjun Jiang

**Affiliations:** The Fifth Medical Center of Chinese PLA General Hospital, Beijing, China

**Keywords:** MSM, HIV/AIDS, Social support, Psychological resilience, Anxiety, Depression

## Abstract

Objective To understand the relationship between psychological resilience in social support and anxiety/depression in people living with HIV/AIDS and to verify whether there is a mediating effect. Methods The questionnaire was administered to 161 people living with HIV/AIDS in a hospital. The questionnaire contained a general questionnaire, the Hospital Anxiety and Depression Scale (HADS), the Psychological Resilience Inventory (CD-RICS), and the Social Collaborative Support Scale (PSSS), and Pearson correlation analyses were used to explore the correlation between the factors and anxiety/depression, stratified linear regression analyses were used to validate the mediation model, and the bootstrap method was used to test for mediating effects. Results Anxiety was negatively correlated with psychological resilience and social support (r=-0.232, P < 0.01; r=-0.293, P < 0.01); depression was negatively correlated with psychological resilience and social support (r=-0.382, P < 0.01; r=-0.482, P < 0.01); there was a mediation effect model of social support between psychological resilience and anxiety/depression; psychological resilience played a fully mediating role in social support and anxiety/depression, with an effect contribution of 68.42%/59.34% and a 95% CI(-0.256~-0.036)/(-0.341 to~-0.106). Conclusion Psychological resilience plays a complete mediating effect between social support and anxiety/depression. It is recommended that more channels of social support be provided to patients with HIV/AIDS, thereby enhancing their psychological resilience and reducing anxiety/depression levels.

## Introduction

HIV continues to be a major global public health issue, having claimed 40.4 million lives so far. In 2022, 630 000 people died from HIV-related causes globally. There were approximately 39.0 million people living with HIV at the end of 2022 with 1.3 million people becoming newly infected with HIV in 2022 globally [1]. In order to end the AIDS epidemic, the Joint United Nations Programme on HIV/AIDS (UNAIDS) has put forward the vision of “ending HIV infection by 2030”, and China has also promulgated the “Thirteenth Five-Year Plan of Action for Containing and Preventing AIDS in China” and the “Implementation Plan for Containing the Spread of AIDS (2019–2022)” [2–4].

With the discovery of epidemiological investigations, there is a significant phenomenon of psychological distress in people living with HIV/AIDS(PLWHA ), especially the state of anxiety and depression [5]. Thus, it is essential to study the psychological characteristics of PLWHA. It was found that the prevalence of depression among PLWHA was 22-44% [6, 7]. While the prevalence of anxiety is 19% [8]. These anxiety and depression problems can affect the effectiveness of antiretroviral therapy and adherence, and increase the transmission and spread of HIV [9]. Consequently, reducing the level of anxiety and depression in PLWHA has been the focus of many researchers. In these studies, it has been established that both psychological resilience and social support are strongly correlated with anxiety/depression, respectively [10–12].

In addition, more in-depth studies have shown that increased levels of social support and psychological resilience can reduce levels of anxiety/depression [13, 14]. At the same time, we found that there is a wider range of studies examining the mental health of PLWHA, and few studies have covered the relationship between psychological resilience and social support with anxiety/depression. We also did not find any similar studies that describe psychological resilience, social support and how they affect anxiety/depression in PLWHA. Therefore, we hope that this study will provide a more in-depth understanding of the psychological world of PLWHA and seek to understand the role and connection between psychological resilience and social support. To be able to provide evidence for a more refined study of the psychological world of PLWHA.

## Methods

### Study design and sample

We are using a cross-sectional research methodology and surveying a specific hospital in Beijing, the capital city of China. In China, information about HIV-infected patients is uploaded to the database of the Chinese CDC, and only specific hospitals are able to receive these HIV-infected patients and administer tests or treatments to them. And it is very appropriate to collect research data in such a specific hospital.

HIV/AIDS patients who attended the HIV outpatient clinic of a hospital in Beijing from January 2023 to August 2023 were selected for the study. Inclusion criteria: (1) Including HIV positive reports; (2) Includes normal cognition, understanding of the study and voluntary participation in cooperating to complete the questionnaire. Exclusion criteria: (1) Including those with significant cognitive impairment or impaired consciousness who could not cooperate in completing the questionnaire; (2) Those who did not want to cooperate in completing the questionnaire for personal reasons.

### Data collection

A standardized-trained psychotherapist from the hospital outpatient clinic introduced the purpose of the study, the principle of confidentiality and related requirements to the patients, and instructed the patients to fill in the questionnaire on a one-to-one basis in strict accordance with standardized procedures. The questionnaire containing the General questionnaire, The Hospital Anxiety and Depression Scale, The psychological resilience scale and The Perceived social support scale was used to collect relevant information. The general questionnaire included demographic characteristics, such as age, education, income, number of sexual partners and occupation.

The Hospital Anxiety and Depression Scale (HADS) was used to measure patients’ anxiety and depression levels. The scale contains 7 questions on the anxiety subscale and 7 questions on the depression subscale, for a total of 14 questions, with a score of 1–4 for each question, and a score of more than 8 for each subscale indicates an abnormality, with higher scores indicating a more pronounced abnormality [15]. The Cronbach’s α coefficients of its total scale, anxiety subscale and depression subscale were 0.879, 0.806, 0.806 respectively, with good reliability and validity [16].

The psychological resilience scale (Connor-Davidson resilience scale, CD-RICS) was used to measure the level of psychological resilience of the patients. The scale contains 13 questions on the resilience subscale, 8 questions on the strength subscale, and 4 questions on the optimism subscale, for a total of 25 questions. Each question is scored 0–4, with higher scores indicating better psychological resilience. Its Cronbach’s alpha coefficient was 0.91, with good reliability and validity [17].

The Perceived social support scale (PSSS) was used to measure the level of social support of patients. The scale contains 4 questions on the family subscale, 4 questions on the friends subscale, and 4 questions on the other subscales, for a total of 12 questions. Each topic is scored 1–7, with 12–36 being low support level, 37–60 being medium support level, and 61 or more being high support level. The Cronbach’s alpha coefficients of its total scale, family subscale, friends subscale, and other subscales were 0.840, 0.818, 0.820, and 0.813, respectively, with good reliability and validity [18, 19].

### Statistical analysis

Analyses were performed using SPSS 26.0 software. Measurements were expressed as ($\stackrel{-}{X}$±S), and t-test or one-way line ANOVA was used to test for differences between groups. Pearson correlation analysis was used to explore the correlation between the factors and anxiety and depression. Stratified linear regression analysis was used to validate the mediation model. The bootstrap method was used to test the mediating effect. P < 0.05 was used to indicate a statistically significant difference.

### Ethics

This study complied with the World Medical Association’s Declaration of Helsinki’s Ethical Principles and Good Clinical Practice for medical research in humans and all applicable regulations.The clinical research protocol and informed consent form were both approved by the Ethics Committee of the Fifth Medical Centre, General Hospital of the Chinese PLA. The subjects recruited for the clinical trial voluntarily signed the informed consent form. (KY-2023-6-41-1).

## Results

### Study participant enrollment and characteristics

The questionnaire was distributed to 170 HIV/AIDS patients, and 161 valid questionnaires were collected, with a recovery rate of 94.71%. The average score of the anxiety scale is 13.72 ± 4.10, and the average score of the depression scale is 15.99 ± 2.58 (Table 1).

**Table 1:** General Information T($\stackrel{-}{X}$±S)

Variable	Constituent ratio (%)	Anxiety		Depression	
		Score	t/F	Score	t/F
Age			0.229		0.279
18~25	16(9.94)	13.35±1.13		14.35±0.50	
26~30	43(26.71)	14.03±0.62		16.95±0.40	
31~40	53(32.92)	13.54±0.52		16.19±0.33	
41~50	31(19.25)	13.42±0.78		15.52±0.42	
>50	18(11.18)	14.40±1.08		15.40±0.72	
Income(RMB)			1.327		0.185
<5 000	51(31.68)	14.53±0.56		16.04±0.39	
5 000~10 000	61(37.89)	13.60±0.54		16.09±0.36	
10 000~15 000	26(16.14)	13.48±0.81		16.02±0.38	
>15 000	23(14.29)	12.56±0.84		15.63±0.43	
Education			1.608		0.143
High school and below	51(31.68)	14.48±0.59		16.16±0.41	
Junior college	38(23.60)	13.94±0.67		15.82±0.43	
Undergraduate course	55(34.16)	13.37±0.55		15.93±0.30	
Graduate student	17(10.56)	12.14±0.81		16.08±0.63	
Number of sexual partners			0.779		0.666
No fixed	84(52.17)	13.41±0.46		15.77±0.31	
1	72(44.72)	13.98±0.48		16.22±0.26	
2 or more	5(3.11)	15.36±0.89		16.44±1.09	
Occupation			0.282		0.615
Student	3(1.9)	12.80±4.99		20.00±1.38	
Production	10(6.2)	15.84±3.30		19.52±3.75	
Sale	18(11.2)	14.53±4.41		18.97±1.77	
Market	7(4.3)	13.36±3.55		18.97±2.05	
Customer Service	10(6.2)	14.74±5.46		18.48±2.90	
Logistics Work	7(4.3)	14.74±5.46		18.62±4.92	
Human Resources	3(1.9)	8.80±0.04		17.60±6.92	
Finance	3(1.9)	15.46±4.80		21.86±3.69	
Civil Service	4(2.5)	11.40±1.36		20.60±1.20	
Technician	12(7.5)	12.33±4.17		19.46±2.34	
Administrators	13(8.1)	13.35±4.01		19.56±2.05	
Teacher	9(5.6)	11.73±3.55		18.40±2.03	
Adviser	1(0.6)	11.20±3.56		20.00±1.22	
Professional	5(3.1)	16.48±6.36		17.12±1.45	
Others	56(34.8)	13.72±4.10		18.84±2.23	

### Analysis of the correlation between psychological resilience, social support, and anxiety/depression

The correlation analysis results indicate that anxiety is negatively correlated with psychological resilience and social support (r=-0.232, P < 0.01; r=-0.293, P < 0.01). Depression is also negatively correlated with psychological resilience and social support (r=-0.382, P < 0.01; r=-0.482, P < 0.01) (Table 2).

**Table 2:** Related analysis(r)

Variable	Anxiety	Depression
Psychological resilience	-0.232^b^	-0.382^b^
Social support	-0.293^b^	-0.482^b^

b:P>0.01

### Testing the mediation effect model

#### Testing the hypothesis of the mediation effect model through layered regression

**Fig. 1:**
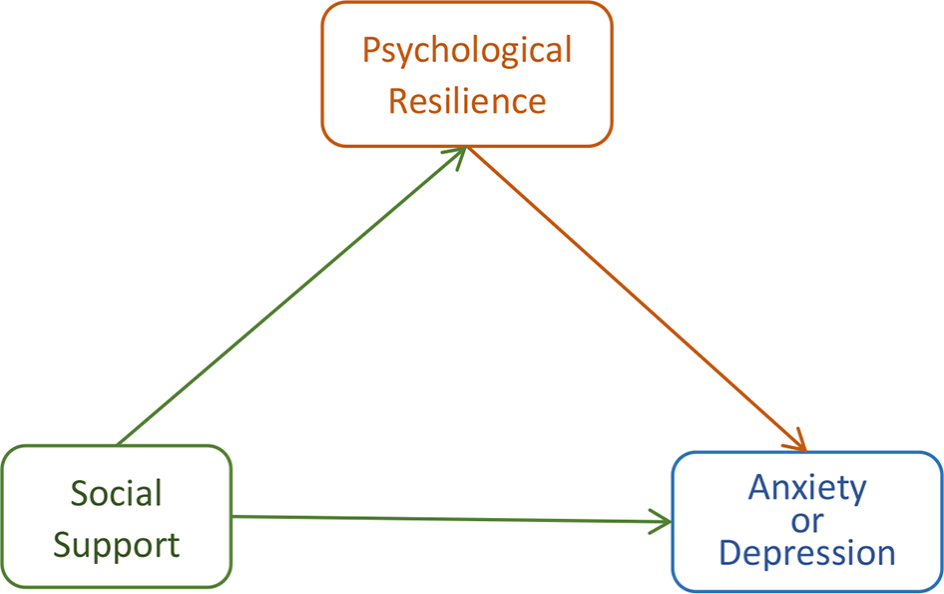
The mediating effect model of psychological resilience between social support and anxiety or depression

A model with social support as the independent variable, psychological resilience as the mediator, and anxiety/depression as the dependent variable was developed (Fig. 1).

Stratified regression analyses were performed when anxiety and depression were the dependent variables, respectively, with control variables (age, income, education, and number of sexual partners) in the first stratum, social support as the independent variable in the second stratum, and mediator variables in the third stratum. The results showed that there was no multicollinearity with VIF>3. In anxiety/depression Eq. 2 (R^2^ = 0.083, P < 0.05/R^2^ = 0.156, P < 0.01), social support was a significant impediment to the level of anxiety/depression (95% CI:-0.135 ~ -0.017, P < 0.01/ 95% CI: -0.127 ~ − 0.056, P < 0.01). With the addition of the mediator variable in anxiety/depression Eq. 3 (R^2^ = 0.120, P < 0.01/R^2^ = 0.257, P < 0.01), psychological resilience was a significant impediment to higher levels of anxiety/depression (95% CI:-0.138 ~ -0.017, P < 0.05/95% CI: − 0.115 ~ -0.046, p < 0.01). The model hypotheses were valid and psychological resilience played a fully mediating role between social support factors and anxiety/depression ( Table 3).

**Table 3:** Stratified regression analyses of anxiety/depression

Variable	95%CI	P	R^2^	ΔR^2^
Anxiety model 1		0.129	0.044	0.044
Age	-0.706 ~ 0.441	0.649		
Income	-1.081 ~ 0.351	0.315		
Education	-1.258 ~ 0.192	0.148		
Number of sexual partners	-0.425 ~ 1.861	0.216		
Anxiety model 2		0.019	0.083	0.039
Age	-0.641 ~ 0.49	0.793		
Income	-0.998 ~ 0.414	0.415		
Education	-1.135 ~ 0.302	0.254		
Number of sexual partners	-0.455 ~ 1.792	0.242		
social support	-0.135~-0.017	0.012		
Anxiety model 3		0.003	0.120	0.037
Age	-0.544 ~ 0.577	0.954		
Income	-1.001 ~ 0.386	0.382		
Education	-1.08 ~ 0.334	0.299		
Number of sexual partners	-0.462 ~ 1.747	0.253		
social support	-0.094 ~ 0.047	0.505		
psychological resilience	-0.138~-0.017	0.012		
Depression model 1		0.657	0.015	0.015
Age	-0.538 ~ 0.194	0.355		
Income	-0.577 ~ 0.337	0.605		
Education	-0.501 ~ 0.425	0.872		
Number of sexual partners	-0.272 ~ 1.187	0.217		
Depression model 2		0.000	0.156	0.141
Age	-0.444 ~ 0.238	0.551		
Income	-0.458 ~ 0.394	0.882		
Education	-0.331 ~ 0.536	0.641		
Number of sexual partners	-0.28 ~ 1.075	0.249		
social support	-0.127~-0.056	0.000		
Depression model 3		0.000	0.257	0.100
Age	-0.332 ~ 0.316	0.960		
Income	-0.449 ~ 0.352	0.812		
Education	-0.261 ~ 0.556	0.477		
Number of sexual partners	-0.268 ~ 1.009	0.253		
social support	-0.078 ~ 0.004	0.073		
psychological resilience	-0.115~-0.046	0.000		

#### Testing for mediating effects via bootstrap

The mediating effects were further tested using the bootstrap method, where the mediating effects model was tested using repeated random sampling 5000 times in the raw data. The results showed that the total effects of anxiety/depression were all significant, none of the direct effects were significant, and none of the confidence intervals for the indirect effects contained 0. Psychological resilience played a fully mediating role in social support factors and anxiety/depression, with an effect contribution of 68.42%/59.34% (Table 4).

**Table 4:** Validation of the mediating role of social support between psychological resilience and anxiety/depression

Variable	Effect	SE	t	P	95%CI
**Anxiety**					
Total effect	-0.076	0.030	-2.554	0.012	-0.135 ~ -0.017
Direct effect	-0.024	0.036	-0.668	0.505	-0.094 ~ 0.047
Indirect effect	-0.052	0.021			-0.097 ~ -0.014
Indirect effects (standardisation)	-0.138	0.057			-0.256 ~ -0.036
**Depression**					
Total effect	-0.091	0.018	-5.092	0.000	-0.127 ~ -0.056
Direct effect	-0.037	0.021	-1.805	0.073	-0.078 ~ 0.004
Indirect effect	-0.054	0.015			-0.083 ~ -0.024
Indirect effects (standardisation)	-0.227	0.060			-0.341 ~ -0.106

## Discussion

Resilience refers to the act of coping, adapting, or thriving from adversity, and reflects a complex and dynamic interplay between individual, environmental, and sociocultural domain [20]. Social support is a social network consisting of three dimensions: family support, friend support, and other support ( such as social relationships with neighbors, leaders, etc.) [21].The level of social support reflects the extent to which an individual is linked to social relationships. The higher the level, the more closely the individual interacts in society [22].

The relationship between psychological resilience, social support, and anxiety/depression is very strong. From the results of this study to observe the relationship between these four factors, an increase in psychological resilience and social support can significantly reduce the level of anxiety/depression, respectively. This result is consistent with the findings of several international studies [23–25]. Among these studies, Leodoro J. Labrague et al.‘s study and Zhi Ye et al.‘s study, although introduced in both social support and psychological resilience can enhance mental health. However, the subjects were healthcare workers and university students, which is different from the target population of this study. While Aneela Hussain et al.‘s study targeted the HIV-infected population and described the importance of social support for mental health. However, none of these studies reported, described the role of psychological resilience in the middle of social support and anxiety/depression.

In the present study, we found that PLWHA who have better scores on psychological resilience and social support mean that they are better able to adapt to being HIV-infected and to survive in society or socialise as HIV-infected people. This adaptation to the environment reduces anxiety or depression due to discrimination or inconvenience of living with HIV infection [26, 27].In looking at PLWHA, Frank H. Galvan concluded that social support is not only an important factor in influencing mental health in addition to the stigma of HIV, but further found a strong relationship between the friend dimension and HIV stigmatization [28].Meanwhile, Cierra N. HOPKINS et al. confirmed the important relationship between psychological resilience and mental health [29]. The results of these two studies are also consistent with some of the results of this study.

In further analysis, we constructed a model of the relationship between psychological resilience, social support, anxiety/depression. The results of the model revealed that social support can directly influence the level of anxiety as well as the level of depression in PLWHA. For a special group of HIV-infected people, the support of family and friends is extraordinarily important [30]. Especially the support of sexual peers. It can even be said that it can influence all aspects of PLWHA, such as how they deal with stigma, whether they take medication as required, and whether they engage in suicidal behaviour [31–33]. Therefore, based on the findings of the study, we suggest that the relevant authorities can pour more resources into family education and sexual peer education for PLWHA.

We found that psychological resilience mediated the effect of social support on anxiety levels or depression levels. Not only can the level of social support directly influence the level of anxiety or depression in PLWHA, but it can also influence the level of anxiety or depression through the strength of psychological resilience. In addition, psychological resilience is an important protective factor for people with low levels of social support and can reduce the occurrence of anxiety and depression [34]. Thus, having good psychological resilience can reduce the occurrence of anxiety or depression at the same level of social support. This provides a strong support in terms of mental health education for PLWHA.

## Strengths and limitations

The results of this study, innovatively confirm, the mediating role of psychological resilience. It also proves how social support and psychological resilience influence anxiety/depression levels in PLWHA. It will tell us a way to a further, more refined understanding of the mental world of PLWHA.

Some limitations of this study that may affect our findings include the small sample size; the data collected may be biased. As the sample was only collected within a single hospital in Beijing, the generalisability of the results must be interpreted with caution. In addition, participant-reported data may have limited the results. Even though we took certain measures to maintain data integrity, it is still not possible to avoid participants’ self-reported data being over- or under-reported. Finally, one of the more unfortunate aspects is the low number of factors for demographic characteristics. This could potentially lead to a number of influencing factors being undetected.

## Conclusion

This study determined the relationship between psychological resilience, social support, anxiety/depression. Social support reduces levels of anxiety or depression in HIV-infected individuals, as does psychological resilience. In addition, psychological resilience is an important mediator between social support and anxiety or depression. Greater psychological resilience prevents the experience of anxiety or depression due to low levels of social support, and mental health work with PLWHA can be more beneficial if it is undertaken in the context of both social support and psychological resilience. Therefore, based on the results of this study, we recommend increased investment in psychotherapy. Mental health judgement, family and peer education by psychotherapists for PLWHA may be a good option [35–37].

## Data Availability

The datasets used and/or analyzed during the current study are available from the corresponding author upon reasonable request.
